# Transvaginal ovarian drilling followed by controlled ovarian stimulation from the next day improves ovarian response for the poor responders with polycystic ovary syndrome during IVF treatment: a pilot study

**DOI:** 10.1186/s12958-019-0559-7

**Published:** 2020-01-24

**Authors:** Bufang Xu, Mingjuan Zhou, Meiyu Cheng, Dan Zhang, Xian Wu, Chenchen Si, Lan Xia, Huihui Xu, Jian Li, Hsun-Ming Chang, Peter C. K. Leung, Aijun Zhang

**Affiliations:** 1 Reproductive Medical Center of Ruijin Hospital, School of Medicine, Shanghai Jiao Tong University, 197 Ruijin 2nd Road, Shanghai, 200025 China; 2 Clinical research center of Ruijin Hospital, School of Medicine, Shanghai Jiao Tong University, Shanghai, China; 3 Department of Obstetrics and Gynaecology, BC Children’s Hospital Research Institute, University of British Columbia, Vancouver, British Columbia Canada; 4 Department of Histo-Embryology, Genetics and Developmental Biology, School of Medicine, Shanghai Jiaotong University, Shanghai Key Laboratory of Reproductive Medicine, 280 South Chongqing Road, Shanghai, 200025 China

**Keywords:** Poor ovarian response, PCOS, TVOD, AMH, Testosterone, BMI

## Abstract

**Background:**

Poor response patients with PCOS who are not susceptible to gonadotropin stimulation are more likely to have canceled cycles or poor clinical outcomes during IVF treatment. However, some limitations exist in the present therapies. In this study, we evaluated the effects of using the transvaginal ovarian drilling (TVOD) followed by controlled ovarian stimulation (COS) from the second day of these poor responders.

**Methods:**

During IVF, 7 poor responders with PCOS and 28 PCOS patients (14 normal and 14 high responders) were recruited. All patients received COS with the gonadotropin-releasing hormone antagonist protocol. For the poor responders, after undergoing 10 to 14 days of ovulation induction with no response, the TVOD was applied and then ovarian stimulation was performed from the next day at the same gonadotropin dose. Serum samples during COS and follicular fluid samples from the dominant follicles on the oocyte pick-up (OPU) day in all three groups were collected. Besides, follicular fluid from small follicles (diameter < 1 cm) in the normal and high responders on the OPU day and those in the poor responders on the TVOD day were gathered. Hormonal levels were examined in all samples using immunometric assays.

**Results:**

All the poor responders restored ovary response after receiving TVOD. There was no significant difference in the stimulation duration, total gonadotrophin dose used and the clinical outcomes among the three groups. The body mass index, serum and follicular levels of anti-Müllerian hormone (AMH) and testosterone in poor responders were higher than those in the other two groups, and the application of TVOD significantly decreased the levels of AMH and testosterone in both serum and follicular fluid.

**Conclusions:**

TVOD followed by ovulation induction from the next day is effective and convenient for poor responders with PCOS. The decline of AMH and testosterone resulted from TVOD may be the main reason resulting in the recovery of ovary sensitivity to gonadotropins. The small sample size is the primary limitation of this study, future studies using a large population cohort and monitoring the long-term outcomes of this strategy will be required.

**Trial registration:**

ChiCTR1900023612. Registered 04 June 2019-Retrospectively registered.

## Background

Polycystic ovary syndrome (PCOS) is the most common endocrine disorder that affects approximately 5–18% of women at reproductive age [1, 2]. PCOS patients generally show outcomes with larger variance in in vitro fertilization and embryo transfer (IVF-ET) treatments compared to normovulatory infertile patients. High responders with PCOS are the patients susceptible to gonadotropin stimulation and produce a large number of follicles with risk of ovarian hyperstimulation syndrome (OHSS) [3]. At the same time, poor responders with PCOS often generate no or only few (< 3) dominant follicles with low serum estrogen (E_2_) levels, even though large gonadotropin doses (≥450 IU/d) are used [4, 5]. Currently, there is a lack of perfect treatment for this group of patients, and most of their IVF cycles had to stop prematurely. In vitro maturation (IVM) is a fertility treatment option for poor responding PCOS patients to controlled ovarian stimulation (COS) [6, 7]. However, the IVM procedure has various limitations and clinical concerns, including lower success rate of implantation, pregnancy, and live birth as well as a higher rate of aneuploidy and miscarriage [8–12]. Ferraretti et al. first applied the transvaginal ovarian drilling (TVOD) and performed COS 2–6 months later for PCOS patients who experienced multiple unsuccessful cycles (cases including OHSS, poor response and poor embryo quality) and found that the ovulation response of these poor responders was restored [5]. However, the shortcomings of their strategy are that the total dosage of gonadotropin used after TVOD increased significantly, and the treatment duration was prolonged for several months. In the present study, we aim to improve the strategy for poor responders with PCOS by performing the COS from the second day after TVOD in the same cycle. Additionally, to understand the mechanism underlying the effect of TVOD on enhancing ovary response, we investigate the serum hormonal levels in patients during COS and the follicle fluid samples collected from small and dominant follicles of the three groups of patients with PCOS (i.e., poor responders, high responders and normal responders).

## Methods

### Patients

Between January 2017 and January 2019, a total of 7 poor response patients with PCOS, aged 24 to 38 years, were selected for the study out of 980 patients with PCOS undergoing treatment with IVF at the Reproductive Medical Center of Ruijin Hospital of Shanghai. The selection criterion was repeated poor response in at least 2 previous IVF cycles (no or <3 dominant follicles developed after two weeks of gradual stimulation with gonadotrophin up to 450 IU [at least 7 days]) [5]. At the same time, another 28 PCOS patients who showed high ovarian response (> 15 dominant follicles developed within two weeks of stimulation, with the E2 level > 4200 pg/ml on the trigger day; *n* = 14.) [13–16] or normal ovarian response (5–15 dominant follicles developed within two weeks of stimulation, with the E2 level < 4200 pg/ml on the trigger day; n = 14.) [13] were recruited as controls. We enrolled women who were diagnosed with PCOS based on the presence of all the Rotterdam criteria [17]. Exclusion criteria included women with previous ovarian surgery and co-existing endocrine diseases (diabetes mellitus, estrogen-dependent tumors, thyroid disease, Cushing’s syndrome, or congenital adrenal hyperplasia). More detailed patient characteristics were analyzed according to the National Institutes of Health (NIH) subclassification of PCOS [2, 18]. All the subjects signed an informed written consent, and the treatment protocol was approved by the Shanghai Jiaotong University Committee on the Use of Human Subjects in Medical Research Institutional Review Board (approval number 2015–92). Prior to COS, patients with hyperandrogenism were treated with cyproterone acetate (CPA) for 1–2 cycles until the decline of serum androgen levels to normal (0.75 ng/ml).

### COS protocol and IVF/ Intracytoplasmic sperm injection (ICSI) procedure

**Fig. 1:**
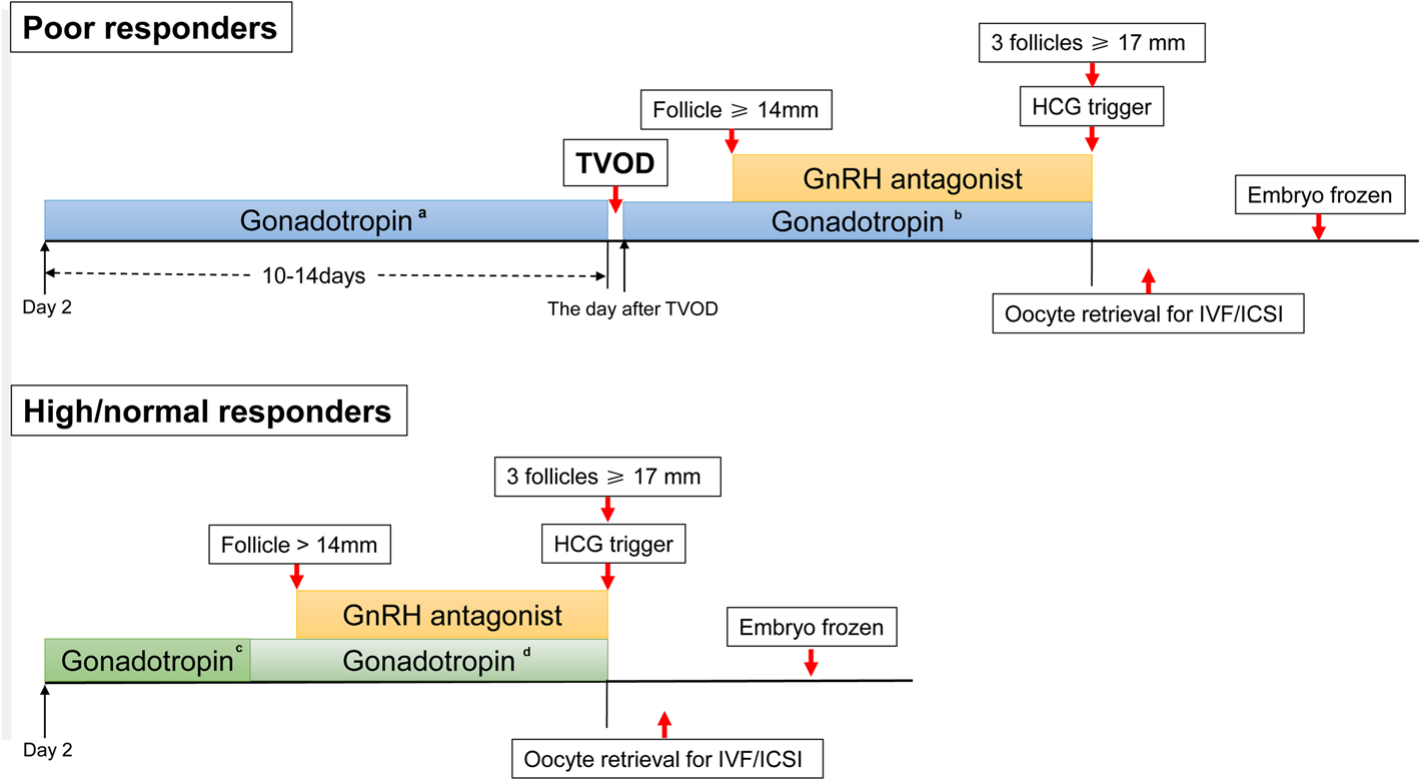
Illustration of the ovarian stimulation protocol in poor responders and in high/normal responders with PCOS. **a**: Maximum gonadotropin dose used in their own previous canceled cycles was selected as the starting gonadotropin dose. **b**: Ovarian stimulation was performed from the next day after TVOD at the same gonadotropin dose previously used for each individual patient. **c**: The starting gonadotropin dose was determined based on the age, AFC, FSH and estrogen levels and BMI. **d**: The gonadotropin dose (from 150 IU/d to 450 IU/d) was adjusted after day 5 of stimulation, depending on the ovarian response, as assessed by the estrogen levels and ultrasound records

All patients received a gonadotropin releasing hormone (GnRH) antagonist protocol. rFSH (Gonal-F, Merck Serono S.A., Switzerland) stimulation was initiated on day 2 of the menstrual cycle. For the normal and high responders, the starting gonadotropin dose was determined according to the age, antral follicle count (AFC), basal follicle stimulating hormone (FSH) and E_2_ levels, and body mass index (BMI). The dose was adjusted after day 5 of stimulation from 150 IU/d to 450 IU/d, depending on the ovarian response, as assessed by the E_2_ levels and ultrasound records. For poor responders, the maximum gonadotropin dose used in their previous cycles (450 IU/d) was selected as the starting gonadotropin dose for the present COS cycle from day 2, and ovarian response was monitored every 5 days. After undergoing 10 to 14 days of ovulation induction without dominant follicles development, the TVOD was applied and ovarian stimulation was performed from the next day at the same gonadotropin dose. All the patients received their daily 0.25 mg cetrorelix acetate (Cetrotide, Merck Serono SA, Switzerland) from the day the leading follicle reached a size of 14 mm onwards up to the trigger day. A total of 3000–7000 IU of hCG (Lizhu, Zhuhai, China) was administered when 3 follicles reached a mean diameter of 17 mm. Oocyte retrieval was performed 35–36 h after hCG injection via transvaginal ultrasound-guided single-lumen needle aspiration (schematic illustration, Fig. 1.). The follicle fluid (3–5 ml) of the first dominant follicle was collected on the day of oocyte retrieval. Moreover, a total of 3–5 ml follicle fluid of small follicles was collected as follows: after extracting the fluid from the dominant follicles, the needles were withdrawn and flushed with cultural medium before puncturing the small follicles (diameter < 1 cm). All the collected follicular fluid samples were centrifuged and stored at − 80 °C for subsequent examinations. Ultrasound examination was performed 2 h later after the oocyte retrieval, and the oocyte volumes of both ovaries were recorded accordingly. Seven days following the oocyte retrieval, ultrasound examination was performed for these patients again when they came back to check the frozen embryos.

ICSI was performed only in cases of severe male factor infertility or previous fertilization failure. Embryo was evaluated and scored 72 h after fertilization according to the previously described criteria [19]. Blastocyst was evaluated and scored according to the criteria presented by Gardner and Schoolcraft (1999) [20]. Given that an extensive duration of stimulation might affect endometrial receptivity among poor responders; all the embryos were frozen before transfer. The other two groups of patients also chose cryopreservation of all the embryos for prevention of hyperstimulation, or because of elevated progesterone levels or poor endometrial morphology or personal reasons. Therefore, all patients in this study underwent the frozen embryo transfer (FET) with a hormone replacement therapy protocol. A maximum of two embryos were transferred into the uterus. All the participants go back to the hospital for the test of blood pregnancy (β-hCG) after 14 days of FET and those women who reveal positive outcomes will make a return visit after 25 and 35 days of FET as well as 12 weeks of gestation to confirm the pregnancy maintenance via ultrasound.

### TVOD

TVOD was performed under general anesthesia with Propofol (Diprivan; Zeneca, Basiglio, Milan, Italy) using a 17-gauge, 35-cm-long needle (K-OPS-1235-Cook IVF, Brisbane, Australia), which was connected to a continuous vacuum pressure system (Craft pump; Rocket Medical, Watford, UK). Approximately 7–8 small follicles (< 1 cm in diameter) were aspirated and scraped under the ultrasound guidance, with approximately 7–8 antral follicles left in each ovary. The whole procedure takes approximately 10 min. A total of 3–5 ml follicle fluid was collected, centrifuged and frozen at − 80 °C for the endocrinal measurement. Patients were followed up with ultrasound and discharged 2–3 h later.

### Measurements of hormones

All follicle fluid and serum samples (500 ul per sample) were centrifuged at 3500 rpm for 10 min and then analyzed at the endocrine laboratory of Reproductive Medical Center of Ruijin Hospital. The hormonal levels were examined using the immunometric assays (the UniCel DxI 800 Access Immunoassay System, Beckman Coulter Diagnostics), with a lower detection limit of 0.2 mIU/mL for FSH, 0.2 mIU/mL for LH, 20 pg/mL for estradiol, 0.1 ng/mL for progesterone, and 0.1 ng/mL for testosterone. The intra-assay and inter- assay coefficients of variance (CV) were 8 and 5.5% for FSH, 5 and 4% for LH, 7 and 10% for estradiol, 3 and 6% for progesterone, and 3 and 3% for testosterone, respectively. AMH levels were determined using an ELISA kit (Guangzhou Kangrun Biological Technology Co., Ltd.), with a lower detection limit of 0.06 ng/ml. The intra-assay and inter-assay coefficients of variance (CV) were 10 and 15%.

### Statistical analysis

For categorical variables, the chi-square was used to compare difference between groups. For continuous variables, the Student *t-*test was performed to compare the difference between two groups and one-way analysis of variance (ANOVA) followed by pairwise comparisons was used for comparisons among 3 groups when the normality (and homogeneity of variance) assumptions were satisfied. In other cases, the Kruskal-Wallis test followed by the Mann-Whitney *U-*test was used to analyze the data. When comparing the difference of serum AMH and testosterone levels on TVOD day and on 6 days later, paired *t* test was applied. Statistical significance was defined as *P* < 0.05.

## Results

### Demographic and clinical characteristics of patients

As shown in Table 1, a total of 16 previous IVF cycles were performed in the 7 poor responders, who had experienced a low ovarian response and had been canceled for the following procedure. Two previous IVF cycles were performed in the high responders, and 1 previous IVF cycle was performed in the normal responders, all of whom received oocyte retrievals with adequate response. There was no significant difference in the demographic characteristics among the three groups on age, basal serum levels of FSH, LH, estradiol, progesterone and PRL. However, the BMI (29.07 ± 2.15 kg/m^2^ vs 22.33 ± 3.39 kg/m^2^ and 23.47 ± 3.76 kg/m^2^, *P* < 0.01) and basal testosterone level before CPA treatment in poor responders (1.02 ± 0.30 ng/ml vs 0.69 ± 0.30 ng/ml and 0.57 ± 0.21 ng/ml, *P* < 0.05) were significantly higher than those in the other two groups. The basal AMH levels in both poor and high responders were higher than that in normal responders (10.94 ± 3.80 ng/ml and 7.50 ± 4.92 ng/ml vs 2.33 ± 2.08 ng/ml*, P* < 0.01), while it was higher in poor responders than which in high responders without significance. The average AFC was higher in poor responders than that in high and normal responders (18.64 ± 1.21 vs 14.36 ± 1.92 and 12.57 ± 0.85, *P* < 0.01). According to the NIH criteria released in 2012, all the 7 poor responders exhibited androgen excess, ovulatory dysfunction and polycystic ovarian morphology (phenotype 1). Therefore, the numbers of phenotype 2 (androgen excess + ovulatory dysfunction), phenotype 3 (androgen excess + polycystic ovarian morphology) and phenotype 4 (ovulatory dysfunction + polycystic ovarian morphology) were 0, 0 and 0, respectively in the poor responders. In the high responders, the numbers of patients belonging to the 4 sub-groups were 10, 0, 0 and 4, respectively. In the normal responders, the numbers of the above 4 phenotypes were 7, 0, 0 and 7, respectively.

**Table 1:** Demographic and clinical characteristics of women with PCOS in the three groups

Variable	Poor responders (*n* = 7)	High responders (*n* = 14)	Normal responders (*n* = 14)	*P* value
PCOS phenotype according to NIH subclassification [2, 18]				
Phenotype 1	7	10	7	–
Phenotype 2	0	0	0	–
Phenotype 3	0	0	0	–
Phenotype 4	0	4	7	–
Age (years)	31.43 ± 1.72	29.86 ± 4.13	30.29 ± 3.31	0.546
BMI (kg/m^2^)	29.07 ± 2.15	22.33 ± 3.39^a^	23.47 ± 3.76^a^	< 0.001
AFC	18.64 ± 1.21	14.36 ± 1.92^ab^	12.57 ± 0.85^a^	< 0.001
Basal FSH level (mIU/ml)	6.45 ± 1.78	6.76 ± 1.79	6.57 ± 1.25	0.904
Basal LH level (mIU/ml)	4.68 ± 2.08	7.17 ± 6.14	4.09 ± 1.80	0.147
Basal estradiol level (pg/ml)	45.86 ± 19.89	45.07 ± 19.93	40.57 ± 15.12	0.747
Basal progesterone level (ng/ml)	0.64 ± 0.28	0.68 ± 0.38	0.63 ± 0.60	0.965
PRL level (ng/ml)	14.64 ± 6.25	20.78 ± 10.92	16.92 ± 5.45	0.239
AMH level (ng/ml)	10.94 ± 3.80^b^	7.50 ± 4.92^b^	2.33 ± 2.08	< 0.001
Testosterone level before CPA treatment (ng/ml)	1.02 ± 0.30	0.69 ± 0.30^c^	0.57 ± 0.21^a^	0.003
Testosterone level after CPA treatment (ng/ml)	0.55 ± 0.14	0.61 ± 0.12	0.57 ± 0.21	0.722
No. of previous stimulated cycles	16	2	1	–
No. of previous canceled cycles	16	0	0	–

Data are presented as mean ± SD. Statistical significance was defined as *P* < 0.05. “a” refers to *P* < 0.01, relative to poor responders. “b” refers to *P* < 0.01, relative to normal responders. “c” refers to *P* < 0.05, relative to poor responders

### Effect of TVOD on poor responders

All the 7 poor responders underwent TVOD did not have any complications, and during the subsequent stimulation cycle, they exhibited a normal ovarian response and underwent oocyte retrieval. As shown in Table 2, the average ovulation stimulation duration was 8.00 ± 1.73 days with a total gonadotrophin dose of 2592.86 ± 430.53 IU, which were similar to those in the other two groups. The number of oocytes retrieved in poor responders had no significant difference to normal responders, while were less than those in high responders (*P* < 0.01). There was no significant difference of the fertilization rate, available embryo rate, embryo implantation rate, clinical pregnancy rate and abortion rate among the three groups. In addition, the endometrial thickness and grade A rate of endometrial morphology on the day of triggering did not significantly differ among these three groups. There was no poor responder experienced severe OHSS. In poor ovarian response patients, the ovarian sizes measured at 2 h and 7 days following oocyte retrieval were similar to those of the normal responders, while the ovarian sizes measured at these time points were lower than those of the high responders.

**Table 2:** Stimulation characteristics of the women with PCOS in the three groups

Variable	Poor responders (*n* = 7) (after TVOD)	High responders (*n* = 14)	Normal responders (*n* = 14)	*P* value
Stimulation duration (days)	8.00 ± 1.73	9.14 ± 0.53	9.21 ± 1.12	0.050
Total gonadotrophin used (IU)	2592.86 ± 430.53	2247.64 ± 1448.42	2140.18 ± 645.08	0.635
No. of oocytes retrieved	11.14 ± 5.55^a^	20.21 ± 4.04	12.79 ± 3.09^a^	< 0.001
Fertilization rate (%)	60/78 (76.9%)	214/283 (75.6%)	145/179 (81.0%)	0.396
No. of available embryos	4.43 ± 2.37	7.43 ± 3.57	4.64 ± 1.95	0.057
Available embryo rate (%)	31/60 (51.7%)	104/214 (48.6%)	65/145 (44.8%)	0.629
Endometrial thickness on the hCG day (cm)	1.06 ± 0.22	0.99 ± 0.17	0.99 ± 0.18	0.721
Grade A rate of endometrial morphology (%)	5/7 (71.4%)	11/14 (78.6%)	8/14 (57.1%)	0.525
Size of left ovary (2 h after oocyte retrieval, cm^3^)	81.64 ± 27.82^a^	184.57 ± 47.89	80.94 ± 12.83^a^	< 0.001
Size of right ovary (2 h after oocyte retrieval, cm^3^)	91.44 ± 29.37^a^	186.10 ± 42.54	87.12 ± 18.33^a^	< 0.001
Size of left ovary (7 days after oocyte retrieval, cm^3^)	78.97 ± 28.90^a^	180.51 ± 34.33	71.10 ± 17.17^a^	< 0.001
Size of right ovary (7 days after oocyte retrieval, cm^3^)	81.31 ± 39.43^a^	173.67 ± 34.67	76.31 ± 17.64^a^	< 0.001
Severe OHSS rate (%)	0/7 (0%)	1/14 (7.1%)	0/14 (0%)	1.000
No. of FET cycles	8	21	23	–
Total number of embryo transfer	15	31	36	–
Cumulative implantation rate (%)	9/15 (60.0%)	15/31 (48.4%)	14/36 (38.9%)	0.371
Cumulative clinical pregnancy rate (%)	8/8 (100.0%)	12/21 (57.1%)	13/23 (56.5%)	0.062
Cumulative abortion rate (%)	1/8 (12.5%)	1/12 (8.3%)	0/13 (0.0%)	0.508

Data are presented as mean ± SD for quantitative variables and frequency for qualitative variables. Size of the ovary was estimated using the equation: π/6 (transverse diameter) × (anteroposterior diameter) × (longitudinal diameter). Statistical significance was defined as *P* < 0.05. “a” refers to P < 0.01, when compared with high responders

As shown in Table 3, we compared the cycles performed after TVOD with the 16 previous cycles performed for the same 7 poor responders. After TVOD, the AFC decreased obviously, and the stimulation duration, total gonadotrophin used per cycle were significantly lower while the maximum estradiol levels, the total number of dominant follicles and the number of oocytes retrieved were higher than those of their previous cycles. The levels of FSH, LH, estradiol, progesterone, AMH and testosterone on stimulation day were not different in previous cycles and in cycles after TVOD.

**Table 3:** Characters of poor responders with PCOS in previous cycle and in cycles after TVOD

Variable	Previous cycles	Cycles after TVOD	P value
No. of stimulated cycles	16	7	–
No. of canceled cycles	16	0	–
Stimulation duration per cycle (days)	14.88 ± 1.59	8.00 ± 1.73	< 0.001
Total gonadotrophin used per cycle (IU)	5608.13 ± 683.80	2592.86 ± 430.53	< 0.001
AFC	19.13 ± 1.35	8.21 ± 0.64	< 0.001
Total number of dominant follicles	4	88	–
Total number of oocytes retrieved	0	78	–
Maximum estradiol level (pg/ml)	181.31 ± 68.91	4340.29 ± 1903.72	0.001
FSH level of stimulation day (mIU/ml)	6.67 ± 1.39	6.45 ± 1.78	0.750
LH level of stimulation day (mIU/ml)	5.48 ± 3.02	4.68 ± 2.08	0.533
Estradiol level of stimulation day (pg/ml)	41.38 ± 14.47	45.86 ± 19.89	0.548
Progesterone level of stimulation day (ng/ml)	0.67 ± 0.21	0.64 ± 0.28	0.802
AMH level of stimulation day (ng/ml)	10.09 ± 3.46	10.94 ± 3.80	0.603
Testosterone level of stimulation day (ng/ml)	0.66 ± 0.18	0.55 ± 0.14	0.157

Data are presented as mean ± SD. Statistical significance was defined as *P* < 0.05

### Hormonal levels in the follicle fluid

As shown in Table 4, the levels of AMH (213.23 ± 85.67 ng/ml vs 12.34 ± 4.08 ng/ml and 13.80 ± 6.77 ng/ml, *P* < 0.01) and testosterone (9.79 ± 4.08 ng/ml vs 6.24 ± 2.62 ng/ml and 4.27 ± 2.54 ng/ml, *P* < 0.05) of small follicular fluid were elevated significantly in poor responders, compared to those in high and normal responders, the PRL level of small follicular fluid in poor responders was lower than those in high responders and normal responders (26.03 ± 14.50 ng/ml vs 56.53 ± 22.39 ng/ml and 48.76 ± 21.95 ng/ml, *P* < 0.05 and *P* > 0.05, respectively). Notably, in the poor responder group, AMH level dramatically decreased from the antral to the dominant follicles after TVOD (from 213.23 ± 85.67 ng/ml to 14.71 ± 8.78 ng/ml, *P* < 0.01). The decreased amplitude was much greater in poor responders compared to that in high (from 12.34 ± 4.08 ng/ml to 9.41 ± 13.13 ng/ml) and normal responders (from 13.80 ± 6.77 ng/ml to 7.33 ± 4.70 ng/ml). Similarly, the small follicles exhibited higher testosterone levels than the ones in dominant follicles in poor responders (9.79 ± 4.08 ng/ml vs 3.42 ± 1.01 ng/ml, *P* < 0.01). However, no significant difference in testosterone levels between small follicles and dominant follicles was found in high (6.24 ± 2.62 ng/ml vs 4.89 ± 1.58 ng/ml, *P* > 0.05) and normal responders (4.27 ± 2.54 ng/ml vs 4.41 ± 1.43 ng/ml, *P* > 0.05). Interestingly, the testosterone level in the poor responders was the lowest in the dominant follicles among the three groups. No significant change was found in follicular fluid PRL levels from small follicles to dominant follicles in all three groups. Moreover, there was no significant difference in other hormonal levels of follicular fluid samples obtained from small and dominant follicles among the three groups.

**Table 4:** Hormonal levels in follicular fluid obtained from small and dominant follicles

	Small follicles (6–10 mm diameter)			Dominant follicles on the OPU day				
Variable	During TVOD in poor responders (*n* = 7)	On the OPU day in high responders (*n* = 14)	On the OPU day in normal responders (*n* = 14)	*P* Value	Poor responders (*n* = 7)	High responders (*n* = 14)	Normal responders (*n* = 14)	*P* Value
FSH (mIU/ml)	9.12 ± 6.77	8.72 ± 4.13	7.17 ± 2.81	0.526	6.27 ± 1.96	7.53 ± 3.31	8.34 ± 4.46	0.480
LH (mIU/ml)	4.03 ± 6.58	4.18 ± 4.15	1.49 ± 2.24	0.199	1.09 ± 0.94	3.29 ± 3.04	2.19 ± 3.09	0.234
Estradiol (pg/ml)	> 9600	> 9600	> 9600	–	> 9600	> 9600	> 9600	–
Progesterone (ng/ml)	15.18 ± 13.51	> 40	> 40	–	> 40	> 40	> 40	–
PRL (ng/ml)	26.03 ± 14.50	56.53 ± 22.39^a^	48.76 ± 21.95	0.013	38.44 ± 21.19	52.95 ± 22.45	53.04 ± 22.96	0.320
AMH (ng/ml)	213.23 ± 85.67	12.34 ± 4.08^c^	13.80 ± 6.77^c^	< 0.001	14.71 ± 8.78 ^c^	9.41 ± 13.13	7.33 ± 4.70^b^	0.270
Testosterone (ng/ml)	9.79 ± 4.08	6.24 ± 2.62^a^	4.27 ± 2.54^c^	< 0.001	3.42 ± 1.01^c^	4.89 ± 1.58	4.41 ± 1.43	0.101

Data are presented as mean ± SD for quantitative variables. Statistical significance was defined as *P* < 0.05. “a” refers to *P* < 0.05, when compared with that in the small follicles of poor responders. “b” refers to *P* < 0.01, when compared with that in the small follicles of normal responders. “c” refers to *P* < 0.01, when compared with that in the small follicles of poor responders

### Serum hormonal levels during COS

**Fig. 2:**
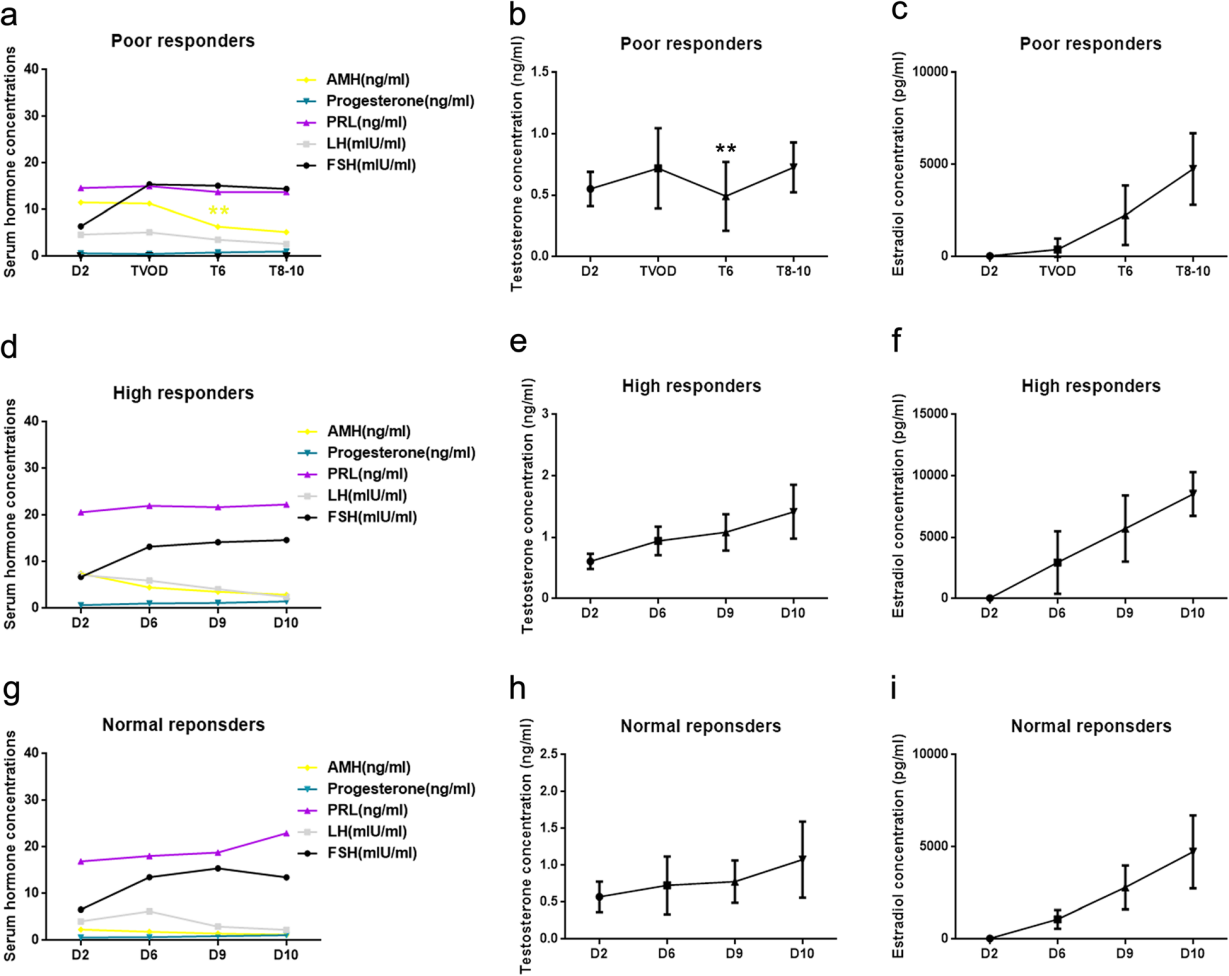
Dynamic changes of serum hormonal levels in poor, high, and normal responders. Serum levels of AMH, progesterone, PRL, LH and FSH in poor responders before and after TVOD **a**. Serum levels of testosterone **b** and estradiol **c** in poor responders before and after TVOD. Serum levels of AMH, progesterone, PRL, LH and FSH in high responders during COS **d**. Serum levels of testosterone **e** and estradiol **f** in high responders during COS. Serum levels of AMH, progesterone, PRL, LH and FSH in normal responders during COS **g**. Serum levels of testosterone **h** and estradiol (**i**) in normal responders during COS. “D2, D6. etc” refers to the second and 6th day. Etc., of the menstrual cycle. “T6 and T8-10” refers to the 6th and 8–10th day after TVOD. Data are presented as mean or mean ± SD. Paired *t* test was applied to calculate the difference of serum AMH and testosterone levels on TVOD day and on 6 days later. ** *P* < 0.01

As shown in Fig. 2, a remarkable decrease in serum levels of AMH and testosterone was observed 6 days after the TVOD in poor responders (*P* < 0.001 and *P* = 0.007, respectively). Notably, after TVOD, the dynamic changes of the hormonal levels in poor responders during COS were similar to those in both normal and high responders. For example, the levels of AMH were substantially declines, whereas the levels of testosterone and estradiol were progressively increased, while PRL had no significant change.

## Discussion

Patients with PCOS respond differently to COS in IVF treatment, a special group of them experience cancellation of treatment cycles or have poor clinical outcomes repeatedly because of poor response with no or very few mature follicles [21]. In the present study, we tried a novel strategy by puncturing the small follicles for these PCOS patients with poor ovarian response followed by COS from the second day and found it was effective and convenient.

In this study, after the poor responders receiving TVOD, both their total gonadotrophin dose used and stimulation duration during the subsequent COS were similar to those in the other two groups. Additionally, the number of oocytes retrieved from poor responders was significantly improved and similar to those in the normal responders with PCOS, suggesting that poor responders with PCOS had become more sensitive to gonadotropin stimulation after ovary drilling. Most importantly, the fertilization rate and available embryo number of poor responders after ovary drilling were similar with those in the other two groups, also the embryo implantation rate, clinical pregnancy rate, and abortion rate in the FET cycles of the poor responder group did not differ from those of the other two groups. All these outcomes suggested that small follicle drilling not only elevate ovary response of poor responders with PCOS, but also have no adverse effects on the quality of oocytes and embryo development during the subsequent COS. It’s worth noting that when compared with the study of Ferraretti et al., TVOD followed with COS instantly shortened the treatment duration, and the total Gonadotropin doses used were much lower (2592.86 ± 430.53 IU vs 3915 ± 1125 IU) [5], suggesting that the ovarian response is improved significantly after ovarian drilling, and TVOD followed with COS instantly is better than starting a new IVF cycle several months later.

Also, our data indicated that obesity along with elevated levels of AMH and testosterone levels in the ovarian microenvironment are the most likely factors that cause the poor ovarian response in patients with PCOS. First of all, in the poor response group, the serum levels of AMH were higher than those in the high and normal groups. We consider that the high AFC and AMH levels in the small follicles were the two main reasons of the high serum AMH levels of the poor responders. Given that the principal function of AMH is the inhibition of the early stages of follicular development and the FSH-dependent selection process [22, 23], we may speculate that the extremely high AMH level in small follicles can induce arrest of follicle development which leads to poor ovarian response to gonadotropins. Secondly, the serum testosterone levels in patients with PCOS decreased to normal levels on the day of ovulation stimulation after treatment with CPA. However, the testosterone levels in the small follicles were still significantly higher than those of the high and normal responders (*P* < 0.05 and *P* < 0.01, respectively). Testosterone can enhance ovarian granulosa cell apoptosis in the antral follicles, which subsequently leads to follicular atresia [24]. Our results suggest that intraovarian hyperandrogenism is most likely another causing factor that induces follicular arrest in women with PCOS [25]. In addition, during the early follicle development, the increased intrafollicular AMH can negatively modulate the shift from the “androgenic” to “estrogenic” by suppressing the activity of aromatase [22]. Thirdly, our results showed that the BMI of poor responders was significantly higher than that in the other two groups (*P* < 0.01), suggesting that obesity (BMI ≥ 28) may be associated with poor ovarian response [26]. The possible reason is that there is a dilution of exogenous FSH in a larger circulating volume [4]. In addition, obesity might be correlated with high serum levels of AMH and hyperandrogenism [27–29]. The PRL levels in the small follicles of poor responders were slightly lower than those in the high responders, suggesting that decreased PRL levels may be associated with a lower ovarian response [30]. It is worth mention that the small follicle fluid of high responders was obtained on the OPU day after receiving the hCG triggering. As hCG has been reported to stimulate the secretion of PRL [31–33], we thus cannot ascertain the regulatory effect of decreased PRL levels on the follicular development in poor responders.

It should be noted that the serum AMH levels in normal responders were relatively low in this study. The possible reason could be as follows: the number of the recruited normal responders (14 cases) was relatively small, which may result in bias. Moreover, studies have shown that AMH values can be influenced by comparable technical, physiological and exogenous factors [34]. Previous studies have shown that the range of AMH levels in PCOS patients is various [35–37], from 0.64 to 50.7 ng/ml [38]. However, these studies did not evaluate the ovarian response in these PCOS patients, we thus hypothesize that the AMH levels in the normal responders may be lower than those in the high responders. We will conduct a retrospective study regarding the various AMH levels in these two groups to confirm our hypothesis. Additionally, the serum testosterone levels of the normal and high responders were slightly lower in this study. As shown in Table 1, 10 of 14 patients with androgen excess in high responders and 7 of 14 patients with androgen excess in normal responders had decreased average testosterone levels. In China, a large number of PCOS patients present the characteristics of oligo- and/or anovulation and polycystic ovaries phenotypes but without clinical (hirsutism) and/or biochemical signs of hyperandrogenism [39, 40]. Certainly, the total testosterone levels rather than the free testosterone levels were analyzed in this study.

Results presented in this study showed that the AFC in poor response patients after TVOD was significantly lower than that of previous cycles (8.21 ± 0.64 vs 19.13 ± 1.35, *P* < 0.001), also AMH levels were markedly decreased in the dominant follicles of poor responders after TVOD, relative to those in the respective small follicles (14.71 ± 8.78 ng/ml vs. 213.23 ± 85.67 ng/ml, *P* < 0.01). Although the AMH levels were also decreased in dominant follicles in both the high and normal responders, it was a greater extent of decrease in poor responders compared to those in the other two groups. Similarly, the degree of testosterone decreased from small follicles to dominant follicles of poor responders was significantly dramatic than those in the other two groups. Correspondingly, results obtained from dynamic changes of serum hormone profile showed that both AMH and testosterone levels decreased immediately after TVOD in poor responders (we consider that the reduced AFC and the decreased levels of the two hormones in these small follicles are the main reasons [41, 42]) (Fig. 2.). All these results suggested that TVOD dramatically reduced the concentrations of AMH and testosterone in ovarian follicles and serum, which subsequently improved the ovarian response to gonadotropins and resulted in successful pregnancy. Some investigators have reported that the endocrine effects of ovarian drilling are rather transient: from days 1 to 5 after laparoscopic ovarian laser evaporation, the decreased hormone levels of testosterone and androstenedione are returned slightly [43]. Accordingly, we assume that the AMH and testosterone levels in local ovarian environment might increase again several months after TOVD, which inhibit the ovarian response, and larger gonadotropin doses would be used when perform COS a few months later.

## Conclusions

In conclusion, TVOD followed by COS from the next day could effectively improve ovarian response for poor responders with PCOS, which is a practical strategy for these patients during their IVF treatment. However, it should be mentioned that because of a low incidence of poor responders in patients with PCOS, the small sample size is the main limitation of this study, future studies using a large population cohort and monitoring the long-term outcomes of this strategy will be required.

## Data Availability

All data generated in the present study are available from the corresponding author on reasonable request.

## Abbreviations

AFC: Antral follicle count
AMH: Anti-Müllerian hormone;
ANOVA: Analysis of variance
BMI: Body mass index
COS: Controlled ovarian stimulation
CPA: Cyproterone acetate
CV: Coefficients of variance
E2: Estrogen
FET: Frozen embryo transfer
FSH: Follicle stimulating hormone
GnRH: Gonadotropin releasing hormone
hCG: Human chorionic gonadotropin
ICSI: Intracytoplasmic sperm injection
IVF: In vitro fertilization
IVM: In vitro maturation
LH: Luteinizing hormone
OHSS: Ovarian hyperstimulation syndrome
OPU: Oocyte pick-up
PCOS: Polycystic ovary syndrome
PRL: Prolactin
TVOD: Transvaginal ovarian drilling
